# Risk factors and predictive model for recurrence in papillary thyroid carcinoma: a single-center retrospective cohort study based on 955 cases

**DOI:** 10.3389/fendo.2023.1268282

**Published:** 2023-09-21

**Authors:** Yin Li, Jiahe Tian, Ke Jiang, Zhongyu Wang, Songbo Gao, Keyang Wei, Ankui Yang, Qiuli Li

**Affiliations:** ^1^ State Key Laboratory of Oncology in South China, Collaborative Innovation Center for Cancer Medicine, Sun Yat-Sen University Cancer Center, Guangzhou, China; ^2^ Department of Head and Neck Surgery, Sun Yat-Sen University Cancer Center, Guangzhou, China; ^3^ Zhongshan School of Medicine, Sun Yat-Sen University, Guangzhou, China

**Keywords:** papillary thyroid carcinoma (PTC), recurrence, initial treatment, non-initial treatment, predictive model, nomogram

## Abstract

**Background:**

The 2015 American Thyroid Association guidelines proposed recurrence risk stratification of differentiated thyroid carcinoma, including papillary thyroid carcinoma (PTC), but this stratification excluded non–initial treatment patients with worse outcomes. This study aimed to explore the potential risk factors for recurrence in PTC and develop a predictive model for both initial and non–initial treatment of patients with PTC.

**Methods:**

A total of 955 patients were included in this study. Differences between the recurrence (−) and recurrence (+) groups were compared. The 955 patients were randomized into two groups: the training group (671 cases) and the validation group (284 cases). All variables were selected using the LASSO regression analysis. A nomogram was developed based on the results of the univariate and multivariate logistic regression analyses. The nomogram performance was evaluated using discrimination and calibration.

**Results:**

Patients aged ≥55 years, extranodal extension (ENE), metastatic LN ratio (LNR) >0.5, and non–initial treatment were identified as potential risk factors for recurrence through LASSO regression and univariate and multivariate analyses. The receiver operating characteristic curve (ROC curve) showed high efficiency, with an area under the ROC curve (AUC) of 0.819 (95% confidence interval [CI], 0.729–0.909) and 0.818 (95% CI, 0.670–0.909) in the training and validation groups, respectively. The calibration curve indicated that the nomogram had a good consistency.

**Conclusion:**

In patients with PTC, age ≥55 years, ENE, LNR >0.5, and non–initial treatment are potential risk factors for recurrence. The predictive model of recurrence was confirmed to be a practical and convenient tool for clinicians to accurately predict PTC recurrence.

## 1 Introduction

Papillary thyroid carcinoma (PTC) is the most common subtype of thyroid carcinoma (TC), and patients with PTC generally have a favorable prognosis (1). However, a subset of patients experience tumor relapse, with recurrence rates ranging from 4.3% to 28% (2, 3). PTC recurrence can complicate surgical resection due to tissue fibrosis and disruption of the normal tissue plane and anatomy, which can increase the likelihood of complications including hypoparathyroidism and injury to the recurrent laryngeal nerve and tracheal cartilage (4). PTC relapse may lead to TC-related deaths, especially among patients experiencing early recurrence, with a mortality rate of 32.3% (5, 6). Therefore, the precise prediction of relapse has become increasingly crucial in guiding treatment decisions and ensuring appropriate patient follow-up.

The 2015 American Thyroid Association (ATA) guidelines proposed risk stratification for recurrence based on molecular markers, which is currently the most effective tool for estimating the possibility of recurrence of differentiated thyroid carcinomas, including PTC (7). Carvalho et al. reassessed the majority of risk factors included in the above stratification and validated the efficiency by dividing the patients into low-, intermediate-, and high-risk groups for recurrence (3). However, with the increased use of high-resolution ultrasonography, more stage I tumors have been identified in newly diagnosed cases over the past decade (8). Changes in PTC patient populations may be related to the characteristics of the thyroid disease. According to the 2022 World Health Organization Classification of Thyroid Neoplasms, molecular subtypes of PTC have been introduced as a new type of classification (9). However, the predictive value of genetic features remains controversial (10). Except these risk factors proposed in the guidelines, recent studies have investigated some lymph node metastasis (LNM)–related factors for predicting patient outcomes, such as the number of metastatic lymph nodes and the metastatic lymph node ratio (11, 12). To date, no prediction model has combined novel risk factors with previously established risk factors from the guidelines.

As the largest cancer center in South China, our hospital is responsible for receiving referred non–initial treatment patients with tumor relapse or insufficient resection scale in previous treatments. In this subset of patients, we collected initial clinicopathological information that was found to be incomplete, making it challenging to predict recurrence. However, non-initial treatment may be associated with increased tumor aggressiveness, advanced tumor stage, and higher recurrence rates (13). The 2015 ATA risk stratification is applicable only to patients undergoing the initial treatment. Although previous studies have investigated risk factors in patients with re-recurrence, there is currently a paucity of research focusing on non–initial treatment patients (11, 13). The risk of recurrence among non–initial treatment patients remains uncertain, and relevant predictive models are not available.

This study aimed to explore the potential risk factors for recurrence in both initial and non–initial treatment patients. Our study also constructed a nomogram of recurrence to assess risk and provide clinicians with a convenient and practical tool for more accurate patient management.

## 2 Materials and methods

### 2.1 Patients

**Figure 1:**
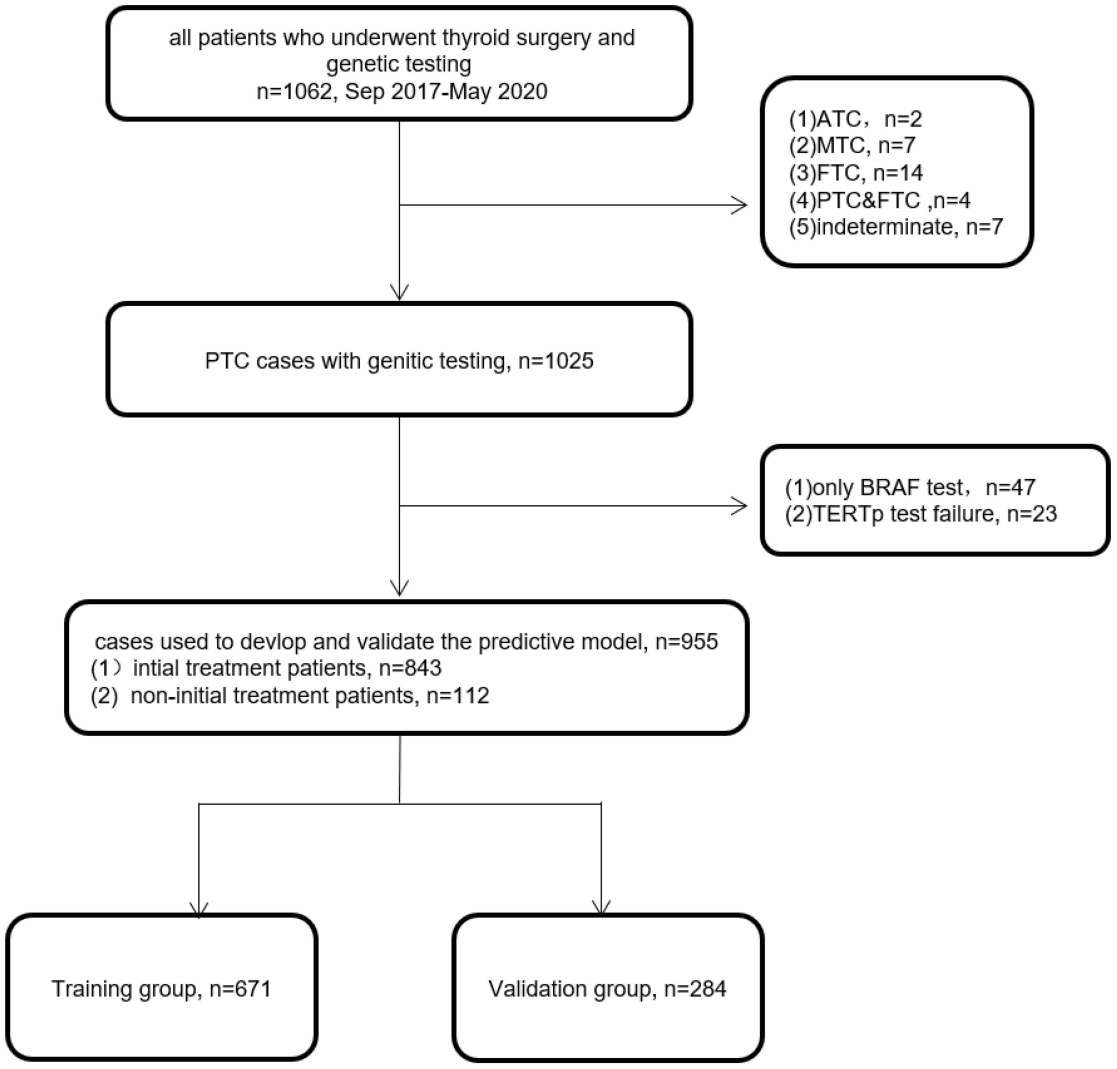
Flowchart of the participants used for model development and validation.

We retrospectively reviewed the medical records of 955 patients with PTC treated at the Department of Head and Neck Surgery between September 2017 and May 2020. This study was approved by the Medical Ethics Committee of the Sun Yat-sen University Cancer Center. The inclusion criteria were as follows: 1) patients who received treatment and genetic testing for PTC at our hospital, and 2) clinical and pathological diagnosis of PTC. The exclusion criteria were as follows: 1) follicular, medullary, or anaplastic TC or 2) PTC without *BRAF V600* or telomerase reverse transcriptase promoter (*TERTp*) mutation status. Finally, a total of 955 patients, including 843 initial treatment patients and 112 non-initial treatment patients, were included in this study. A flowchart of the participant selection process is presented in **Figure 1**. Patients were randomly divided into two groups (at a ratio of 7:3): training group (n = 671) and validation group (n = 284). Statistical power was used to assess the reliability of the study, considering the sample sizes and research outcomes in the two sets (14).

The patient list was filtered and imported from our hospital’s big data platform. Clinical baseline characteristics were retrospectively collected from the His Medical System. The pathological characteristics of PTC were collected according to the AJCC 8th edition/TNM classification system and 2015 ATA guidelines. The genetic testing results included *BRAF V600* and *TERTp* mutations. The cutoff values for LNR and age were set according to relevant studies and the AJCC 8th edition TNM classification system, respectively (15, 16). The initial treatment group included patients who had complete pathological information and underwent initial surgery at our center. The non-initial treatment group included patients who had undergone initial surgery in other hospitals. Clinicopathological information of non-initial treatment patients was mainly based on reoperation specimens in our hospital. Recurrence was defined as new pathologically confirmed PTC (fine-needle aspiration or surgical pathology) or radiographically confirmed distant metastases. The pathological subtypes of PTC were divided into two groups based on their aggressiveness. The low-aggressive subtypes included classical and follicular variants. The highly-aggressive subtypes include tall cell, columnar cell, solid, diffuse sclerosing, and hobnail (7).

### 2.2 Statistical analysis

We applied least absolute shrinkage and selection operator (LASSO) regression analysis to reduce the number of risk factors with the best predictive performance using tenfold cross-validation based on the “glmnet” package in R. These factors were further filtered through univariate logistic regression analysis. The potential variables with *P <*0.05 were considered statistically significant and were used to perform multivariate logistic regression. A nomogram was built based on the risk factors identified in the multivariate analysis. The performance and accuracy of this recurrence prediction model were estimated and identified in the training and validation groups. The predictive ability of this model was evaluated using receiver operating characteristic (ROC) curve and area under the ROC curve (AUC). The predictive accuracy of the model was evaluated using calibration curves and the Hosmer–Lemeshow test. The results were plotted using bootstrapping with 1,000 resamples.

Statistical analyses were performed using SPSS (version 26, Chicago, IL, USA) and R software (version 4.2.1, http://www.Rproject.org). Classification data were expressed as percentages, and means ± standard deviations were described as continuous variables. Odds ratios (ORs) were calculated using univariate and multivariate logistic regression of the variables. The Student’s t-test or Mann–Whitney U test was used to compare continuous variables with baseline data, and the chi-square or Fisher’s exact test was used for categorical variables. All patients were randomized into two groups using the “carret” package. LASSO was performed using the “glmnent” package. ROC curves were plotted using the “pROC” package. The nomogram and calibration curves were plotted using the “RMS” package. Statistical significance was set at p-value of <0.05.

## 3 Results

### 3.1 Comparison of patients’ clinicopathological characteristics between recurrence (−) and recurrence (+)

The baseline patient characteristics are presented in **Table 1**. Overall, 955 cases of PTC were included and treated at our cancer center between 2017 and 2020. The overall recurrence rate was 3.98% (38/955). All characteristics were compared between recurrence (−) and recurrence (+) groups. A higher positive rate of *TERTp* mutation (13.16% vs. 2.29%, *P <*0.001) and the proportion of non-initial treatment patients (55.26% vs. 9.92%, *P <*0.001) were observed in the recurrence (+) group. Recurrence (+) was significantly associated with a higher proportion of patients aged >55 years (*P <*0.001), extrathyroidal extension (ETE; *P <*0.001), ENE (*P <*0.001), radioiodine (RAI) treatment (*P* = 0.003), more advanced tumor stage (*P* = 0.005), higher metastatic LN rate (LNR) >0.5 (*P <*0.001), lower incidence of coexisting Hashimoto’s thyroiditis (HT; *P* = 0.037), and nodular goiter (NG; *P* = 0.048). There were no significant differences in sex (*P* = 0.712), average age (*P* = 0.479), vascular invasion (*P* = 0.340), number of metastatic LNs (*P* = 0.062), *BRAF V600* mutation (*P* = 0.210), or aggressive subtypes (*P* = 0.868) between the two groups. Clinicopathological characteristics of the training and validation groups are shown in **Table 2**. There was no significant difference existed in the levels of these variables between the two groups among most factors, indicating good consistency between the two groups.

**Table 1:** Comparison of clinicopathological characteristics of patients between recurrence (-) and recurrence (+).

Variables	Recurrence (−) (N = 917)	Recurrence (+) (N = 38)	P-value
Gender, No. (%)			0.712
female	618 (67.39%)	24 (63.16%)	
male	299 (32.61%)	14 (36.84%)	
Age (M± SD, years)	39.878 ± 11.568	41.263 ± 17.042	0.479
<55 years	808 (88.11%)	26 (68.42%)	0.001
≥55 years	109 (11.89%)	12 (31.58%)	
HT (%)			0.037
No	681 (76.17%)	35 (92.11%)	
Yes	213 (23.83%)	3 (7.89%)	
NG (%)			0.048
No	418 (48.44%)	24 (66.67%)	
Yes	445 (51.56%)	12 (33.33%)	
Vascular invasion (%)			0.340
No	901 (98.26%)	36 (94.74%)	
Yes	16 (1.74%)	2 (5.26%)	
ETE (%)			<0.001
No	730 (79.61%)	21 (55.26%)	
Yes	138 (15.05%)	8 (21.05%)	
^*^Unknown	49 (5.34%)	9 (23.68%)	
Number of metastatic LNs, No. (%)			0.062
≤5 metastatic LNs	670 (73.06%)	22 (57.89%)	
>5 metastatic LNs	247 (26.94%)	16 (42.11%)	
BRAF V600, No. (%)			0.210
negative	241 (26.28%)	14 (36.84%)	
positive	676 (73.72%)	24 (63.16%)	
TERT, No. (%)			<0.001
negative	896 (97.71%)	33 (86.84%)	
positive	21 (2.29%)	5 (13.16%)	
Subtypes, No. (%)			0.868
low aggressive	908 (99.02)	37 (97.37)	
high aggressive	9 (0.98)	1 (2.63)	
ENE, No. (%)			<0.001
No	853 (93.02%)	27 (71.05%)	
Yes	64 (6.98%)	11 (28.95%)	
RAI treatment, No. (%)			0.003
No	868 (94.66%)	31 (81.58%)	
Yes	49 (5.34%)	7 (18.42%)	
^$^Stage, No. (%)			0.005
I + II	898 (97.93%)	34 (89.47%)	
III + IV	19 (2.07%)	4 (10.53%)	
LNR, No. (%)			<0.001
≤0.5	796 (86.80%)	25 (65.79%)	
>0.5	121 (13.20%)	13 (34.21%)	
Initial Treatment, No. (%)			<0.001
Yes	826 (90.08%)	17 (44.74%)	
No	91 (9.92%)	21 (55.26%)	

^*^ETE (Unknown): regional lymph node positive but the tumor was removed in previous surgery; ^$^Stage: according to the adjusted AJCC 8th guideline.

HT, Hashimoto’s thyroiditis; NG, nodular goiter; ETE, extrathyroidal extension; ENE, extranodal extension; RAI treatment, radioiodine treatment; LNR, metastatic lymph node ratio.

**Table 2:** Clinicopathological characteristics of the training and validation groups.

Variables	Training group (N= 671)	Validation group (N=284)	P value
Gender, No. (%)			0.950
female	452 (67.36%)	190 (66.90%)	
male	219 (32.64%)	94 (33.10%)	
Age (M± SD, years)	40.082 ± 11.689	39.581 ± 12.151	0.550
<55 years	587 (87.48%)	247 (86.97%)	0.912
≥55 years	84 (12.52%)	37 (13.03%)	
HT (%)			0.017
No	517 (79.05%)	199 (71.58%)	
Yes	137 (20.95%)	79 (28.42%)	
NG (%)			0.326
No	301 (48.01%)	141 (51.84%)	
Yes	326 (51.99%)	131 (48.16%)	
Vascular invasion (%)			0.939
No	659 (98.21%)	278 (97.89%)	
Yes	12 (1.79%)	6 (2.11%)	
ETE (%)			0.126
No	534 (79.58%)	217 (76.41%)	
Yes	93 (13.86%)	53 (18.66%)	
^*^Unknown	44 (6.56%)	14 (4.93%)	
Number of metastatic LNs, No. (%)			0.103
≤5 metastatic LNs	497 (74.07%)	195 (68.66%)	
>5 metastatic LNs	174 (25.93%)	89 (31.34%)	
BRAF V600, No. (%)			0.670
negative	176 (26.23%)	79 (27.82%)	
positive	495 (73.77%)	205 (72.18%)	
TERT, No. (%)			0.592
negative	651 (97.02%)	278 (97.89%)	
positive	20 (2.98%)	6 (2.11%)	
Subtypes, No. (%)			1.000
low aggressive	664 (98.96%)	281 (98.94%)	
high aggressive	7 (1.04%)	3 (1.06%)	
ENE, No. (%)			0.400
No	622 (92.70%)	258 (90.85%)	
Yes	49 (7.30%)	26 (9.15%)	
RAI treatment, No. (%)			0.391
No	635 (94.63%)	264 (92.96%)	
Yes	36 (5.37%)	20 (7.04%)	
^$^Stage, No. (%)			0.761
I + II	656 (97.76%)	276 (97.18%)	
III + IV	15 (2.24%)	8 (2.82%)	
LNR, No. (%)			0.943
≤0.5	576 (85.84%)	245 (86.27%)	
>0.5	95 (14.16%)	39 (13.73%)	
Initial Treatment, No. (%)			0.134
Yes	585 (87.18%)	258 (90.85%)	
No	86 (12.82%)	26 (9.15%)	
Recurrence, No. (%)			0.942
No	645 (96.13%)	272 (95.77%)	
Yes	26 (3.87%)	12 (4.23%)	

^*^ETE (Unknown): regional lymph node positive but the tumor was removed in previous surgery; ^$^Stage: according to the adjusted AJCC 8th guideline.

HT, Hashimoto’s thyroiditis; NG, nodular goiter; ETE, extrathyroidal extension; ENE, extranodal extension; RAI treatment, radioiodine treatment; LNR, metastatic lymph node ratio.

### 3.2 LASSO, univariate, and multivariate logistic regression analyses

**Figure 2:**
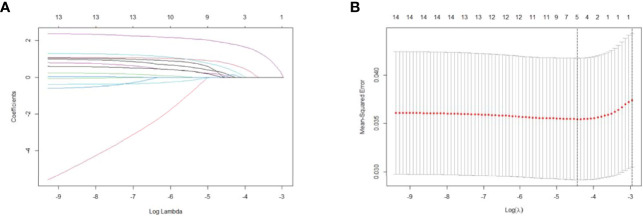
Selection of significant parameters for clinicopathological variables in the training set. **(A)** LASSO coefficient profiles. The y axis represents the coefficient values of the features. The x axis represents the log(λ) value. As the value of log(λ) increased, the degree of model compression and the function of the model to select important variables also increased. **(B)** Cross-validation for tuning parameter selection in the LASSO model. The value in the middle of the two dotted lines is the range of the positive and negative standard deviations of the log(λ). The dotted line on the left indicates the value of the harmonic parameter log(λ) when the error in the model is minimized. Six variables were selected when log(λ) = −4.4. LASSO, least absolute shrinkage and selection operator.

**Figure 3:**
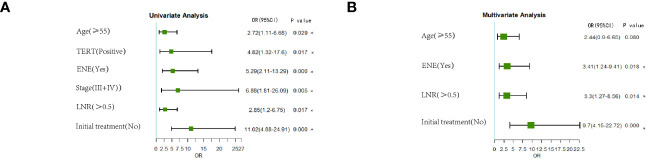
Forest plot of univariate and multivariate logistic regression analyses **(A, B)**. *P<0.05.

LASSO regression analysis included potential risk factors defined in the 2015 ATA guidelines and previous studies. We observed that non-initial treatment may be associated with a higher recurrence rate despite receiving comprehensive therapy including treatment with surgery, RAI, and thyroid-stimulating hormone suppression during clinical practice. Therefore, initial or non-initial treatment was included as a potential risk factor for recurrence. Six predictors were selected from the 15 candidates through LASSO regression, including age >55 years, *TERTp* mutation, ENE, stage, LNR >0.5, and non-initial treatment (**Figures 2A, B**). The significant risk factors in the training group were further selected through univariate and multivariate logistic regression analyses (**Figures 3A, B**). Multivariate logistic analysis showed that age ≥55 years (OR, 2.44; 95%CI 0.9–6.65; *P* = 0.080), ENE (OR, 3.41; 95%CI 1.24–9.41; *P* = 0.018), LNR >0.5 (OR, 3.3; 95%CI 1.27–8.56; *P* = 0.014), and non-initial treatment (OR, 9.7; 95%CI 4.15–22.72; *P <*0.001) were associated with recurrence.

### 3.3 Development and validation of the nomogram

**Figure 4:**
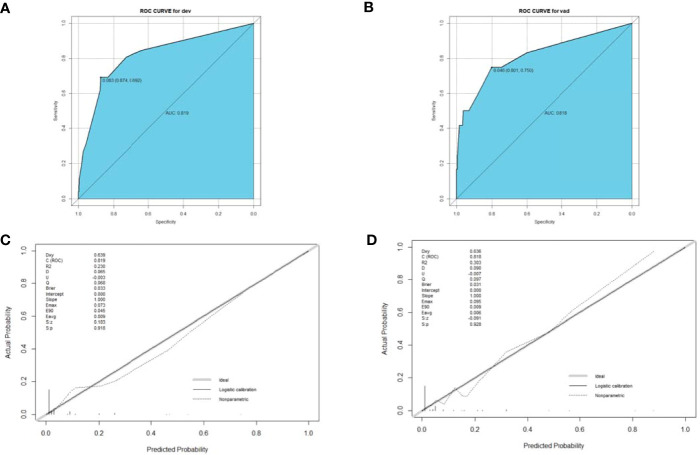
ROC curve **(A, B)** and calibration curve **(C, D)** for the training and validation groups. The ROC curve based on potential risk factors identified by multivariate logistic regression analysis showed a great ability to distinguish recurrence in both the training and validation groups with a high AUC value **(A, B)**. The calibration curve indicated good consistency in the training **(C)** and validation groups **(D)**. ROC, receiver operating characteristic; AUC, area under the curve.

**Figure 5:**
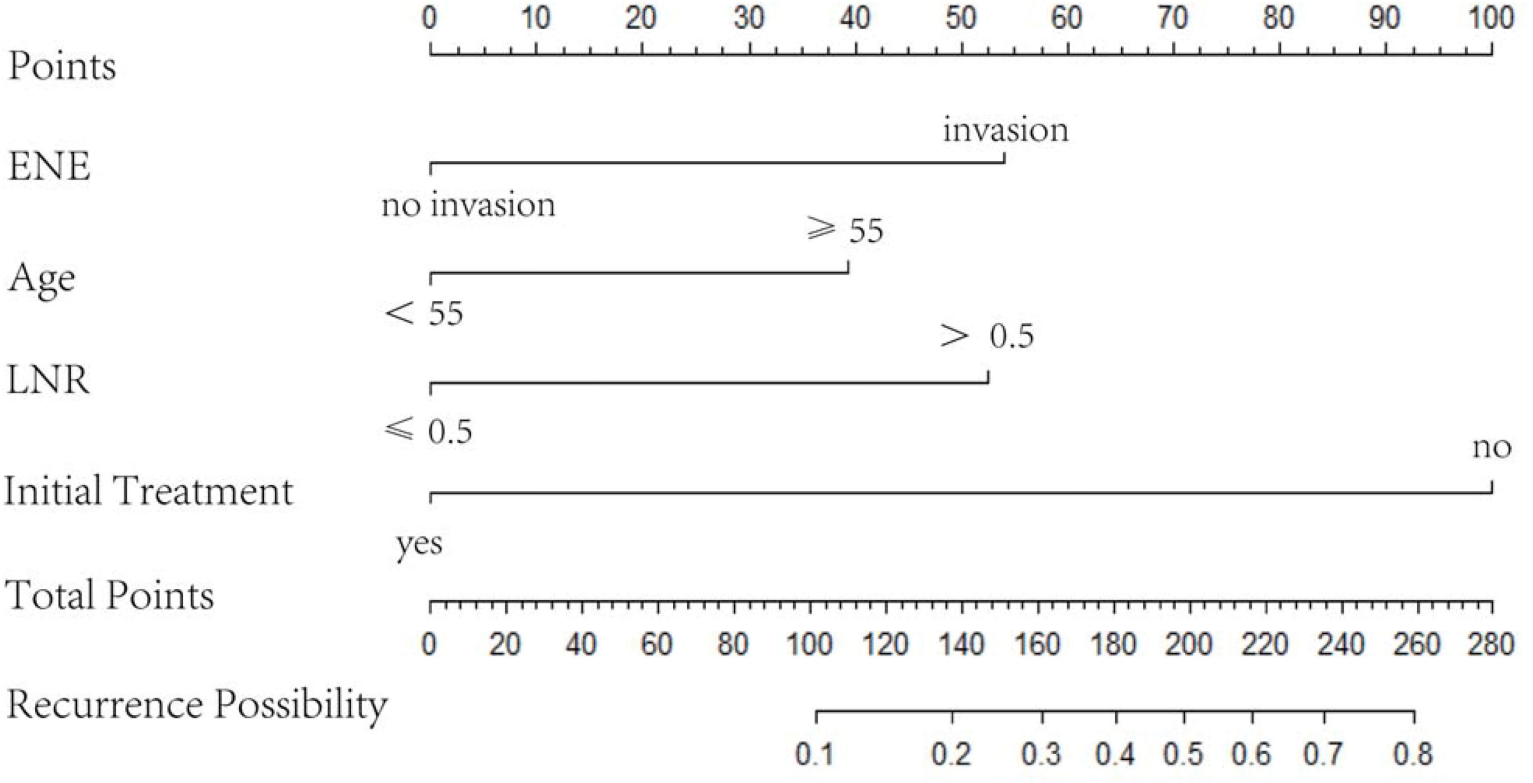
Nomogram based on four variables, namely age ≥55 years, ENE, LNR, and non-initial treatment. LNR, metastatic LN ratio; ENE, extranodal extension.

A nomogram was established based on the results of the multivariate logistic regression. An ROC curve was drawn to evaluate the diagnostic effectiveness of the model (**Figure 4**). The AUC of the nomogram was 0.819 (95%CI, 0.729–0.909), with specificity of 87.4% and a sensitivity of 69.2% in the training group (**Figure 4A**). In the validation group, the AUC of the nomogram was 0.818 (95%CI, 0.670–0.909) with a specificity of 80.1% and a sensitivity of 75%, and the results are shown in **Figure 4B**. The model was evaluated by constructing calibration curves. Calibration curves (**Figures 4C, D**) and the Hosmer–Lemeshow test indicated good consistency between the nomogram-predicted probability of recurrence and the actual recurrence rate in both the training and validation groups (*P* = 0.9883 and *P* = 0.9998). To visualize the model, we plotted the nomogram of our predictive model based on four factors: age ≥55 years, ENE, LNR >0.5, and non-initial treatment (**Figure 5**). Every variable was scored by drawing a straight line upward the “points” line. The total points are the sum of the points obtained by the four factors. A straight line down to the axis named “recurrence possibility” represents the risk of recurrence. For individual patients with PTC, the risk of recurrence was calculated using this nomogram.

## 4 Discussion

The application value of the 2015 ATA risk stratification in initial treatment patients has been well substantiated, and some studies have confirmed the relevant risk factors for patients with re-recurrence (13, 17). However, there is currently a dearth of research exploring relapse risk, especially among non-initial treatment patients in comparison with their initial-treatment counterparts. Compared with previous studies, our study makes some improvements. In this study, we included both initial treatment and non–initial treatment patients and reassessed the predictive value of these risk factors in this study cohort. In addition to investigating the independent risk factors, we proposed a predictive model to assess the accuracy and effectiveness of this model.

In this study, initial treatment was the most important predictive factor for recurrence. Recurrence in patients often results in a referral to a higher-level hospital for further treatment, leading to incomplete patient information and exclusion from retrospective studies (18, 19). In contrast, in this study, non–initial treatment patients were more likely to relapse and accounted for 55.26% of all patients with recurrence. Thus, including this population may have changed the previously favorable impression of PTC prognosis. The high recurrence rate in this population may be due to the following reasons: 1) Limited sensitivity of imaging examinations may result in more occult LNM and higher lymph node recurrence rates (20, 21). 2) Due to insufficient experience in treating complex cases of PTC, failure to perform en block resection or R1/R2 resection may increase the risk of recurrence (22). 3) Irregular postoperative follow-up may delay the detection of recurrence and increase the risk of multiple recurrences owing to favorable prognosis (23). Therefore, the treatment strategy for non-initial treatment patients should be made by experienced and senior surgeons, and a more aggressive resection extent should be considered. This subset of patients should undergo active surveillance.

Some studies have indicated that the prognostic significance of *BRAF V600* in terms of recurrence might not be robust. Several studies, including those conducted by Xing et al. and Shen et al., had reported the correlation between *BRAF V600* mutation and the high rate of recurrence and mortality in patients with PTC. Shen et al. specifically reported a linear relationship between patient age and mortality in patients with the *BRAF V600* mutation; however, this significance disappeared after adjusting for factors such as LNM, ENE, and distant metastasis (24, 25). Additionally, studies conducted in East Asian countries, including Taiwan, Korea, and Japan, have demonstrated that *BRAF V600* mutation is not necessarily a poor prognostic factor (26–28). Our study showed that *BRAF V600* mutation did not represent a difference between recurrence (+) and recurrence (−). In addition, the *TERTp* mutation was identified as a risk factor for recurrence both in our study and in previous studies, but its low positive rate resulted in its exclusion from the final risk-stratification model (29). Consequently, genetic testing for PTC may have limited clinical value for specific patient subgroups and should not be considered as a routine indicator for postoperative surveillance.

ENE and LNR have been identified as adverse prognostic factors in previous studies (3, 11). In this study, these nodal factors were associated with recurrence, which is consistent with the findings of previous studies. Non-initial treatment patients lack information regarding their initial LNM status, which may affect the accuracy of prognosis predictions based solely on the number of metastatic LNs (12). As such, LNR and ENE may serve as more appropriate indicators for assessing the risk of recurrence. An increasing number of studies have identified that the coexistence of HT or NG is associated with an increased risk of developing TC but may be a protective factor against PTC progression and metastasis. Our study revealed a significant difference in the coexistence of HT or NG between recurrence (+) and recurrence (−). Previous findings have suggested that HT and NG may restrict tumor progression through a certain mechanism and improve patient outcomes. A high proportion of tumor-infiltrating lymphocytes could explain the protective role of HT; however, the same effect of NG remains uncertain (30, 31).

The proposed predictive model exhibits the ability to accurately prognosticate based on existing patient characteristics, thereby surpassing the limitations of conventional research. Moreover, this model highlighted the risk of relapse among non-initial treatment patients, potentially attributable to comprehensive pathological and socioenvironmental factors. Thus, clinicians should reassess their optimistic stance on the favorable prognosis of PTC in non-primary cases, necessitating a more proactive and cautious approach for the diagnosis and management of such patients.

This study has some limitations. First, all enrolled patients were recruited from the same cancer center without external validation. Moreover, we need to expand the sample size to reduce heterogeneity. In addition, the data were collected retrospectively and remained open to an unknown selection bias. Furthermore, some important risk variables, such as the LNM dimensions were not assessed in this study. Finally, the follow-up time may be too short, as some patients with a low recurrence risk may manifest clinically significant recurrence many years after treatment.

## 5 Conclusion

To the best of our knowledge, this study is the first to investigate the risk factors for recurrence in both initial treatment and non–initial treatment patients. Compared to existing tools, we developed a simplified and efficient prognostic predictive model based on the following risk factors: age ≥55 years, ENE, LNR >0.5, and non-initial treatment. The identification of these risk factors fills a critical gap in the prognostic tools for the non-initial treatment population. This study will provide useful guidance for individualized treatment and follow-up of patients with PTC, especially for non–initial treatment patients.

## Data availability statement

The original contributions presented in the study are included in the article/supplementary material. Further inquiries can be directed to the corresponding author.

## Ethics statement

The studies involving humans were approved by the Medical Ethics Committee of the Sun Yat-Sen University Cancer Center. The studies were conducted in accordance with local legislation and institutional requirements. The ethics committee/institutional review board waived the requirement for written informed consent for participation from the participants or the participants’ legal guardians/next of kin because written informed consent for participation was not required for this study in accordance with national legislation and institutional requirements.

## Author contributions

YL: Data curation, Formal Analysis, Software, Writing – original draft. JT: Writing – original draft, Data curation, Software. KJ: Data curation, Writing – original draft. ZW: Methodology, Software, Writing – original draft. SG: Methodology, Software, Writing – original draft. KW: Data curation, Methodology, Writing – original draft. AY: Conceptualization, Writing – review & editing. QL: Conceptualization, Writing – original draft, Writing – review & editing.
